# Response of the bGeigie Nano and CzechRad Monitors to Secondary Cosmic Radiation

**DOI:** 10.3390/s24247915

**Published:** 2024-12-11

**Authors:** Petr Kuča, Jan Helebrant, Peter Bossew

**Affiliations:** 1 National Radiation Protection Institute (SÚRO), 140 00 Praha, Czech Republicjan.helebrant@suro.cz (J.H.); 2 Graduate School of Health Sciences, Hirosaki University, Hirosaki 036-8560, Japan

**Keywords:** bGeigie Nano, CzechRad, G-M detector, secondary cosmic radiation

## Abstract

Ambient dose rate surveying has the objective, in most cases, to quantify terrestrial radiation levels. This is true in particular for Citizen Monitoring projects. Readings of detectors, which do not provide spectrally resolved information, such as G-M counters, are the sum of contributions from different sources, including cosmic radiation. To estimate the terrestrial component, one has to subtract the remaining ones. In this paper, we investigate the cosmic response of two particular monitors, the bGeigie Nano, which has been used extensively in the Safecast Citizen Monitoring project, and its upgraded version, the new CzechRad, which uses the same G-M detector, and show how the local contribution of cosmic radiation can be estimated.

## 1. Introduction

Citizen Science (CS) means scientific research conducted by citizens who are not professional scientists. Their involvement can range between participating to different degrees in defined projects and setting up entire projects. Citizen monitoring (CM), as a discipline in CS, focuses on data acquisition and is a potentially extremely powerful way to monitor and survey environmental quality regarding pollution due to anthropogenic routine or accidental activity, or the natural background. For a large compendium of CS across fields of science, see [1] and the very comprehensive Wikipedia entry about CS, https://en.wikipedia.org/wiki/Citizen_science (accessed on 4 November 2024).

A very successful CM project is Safecast [2] (https://blog.safecast.org/ accessed on 4 November 2024). Among others, this platform enabled creating a world-wide map of ambient dose rate (ADR) through contributions of numerous citizens, still expanding, who submit data which they have acquired using a standard monitor (Currently, October 2024, the map is not active). The data and methodology are openly accessible. The project was founded shortly after the Fukushima accident 2011 in Japan, motivated by the perceived mistrust in official information policy, and soon spread world-wide. Several thousand citizens contribute as volunteers, carrying detectors and sending data to Safecast.org, where they are presented in a freely accessible map. The project uses a standard device called bGeigie Nano (based on a G-M detector), which is the subject of investigation in this paper, but in principle, other monitors of similar type can be used.

Among the benefits of CM is its ability to generate large amounts of data, its contribution to democratization of science and its educative potential. On the other hand, CM is mostly performed by people who are not metrological professionals. This causes QA problems, in particular related to the notions of representativeness, observation protocol and the interpretability of results.

This paper aims to one particular aspect of interpretability. Most dose rate monitors, especially those based on G-M detectors, do not provide spectral information of incident radiation. The reading of the instrument represents the sum of several components, namely, terrestrial radiation, cosmic radiation, airborne radiation and internal background. Usually in area monitoring, one is interested only in the geographical variability in terrestrial radiation; in this case, one step in the task of data interpretation consists in trying to subtract the other components.

If no independent measurement of these components is available, which is the most common case, one has to estimate them from other information. Here, we concentrate on estimation of the cosmic component, but also discuss the internal background and contribution of airborne radon progeny, because they play a role in assessing the response of the device to cosmic radiation.

In the rather long methods Section 2, we start with some basics on cosmic radiation (Section 2.1), followed by discussion of the response of G-M detectors to cosmic radiation in general (Section 2.2) and factors that influence the intensity of cosmic radiation (Section 2.3), followed by a description of the bGeigie Nano and CzechRad monitors (Section 2.4). Next, in Section 2.5, components of radiation registered by the monitor are discussed in more detail. Finally, in Section 2.6, we propose two methods for assessing cosmic response. Results from the application of these methods are presented in Section 3.

This paper builds on the practical experience of the authors in working with the bGeigie Nano and CzechRad devices and deepens information which in lesser detail has been incorporated in previous publications, such as [3,4,5,6,7,8,9].

## 2. Materials and Methods

### 2.1. Secondary Cosmic Radiation

Cosmic rays originate from galactic and intergalactic space and to a smaller extent from the sun. They consist mainly of protons and a small fraction of heavier nuclei. Interaction with the atmosphere results in secondary cosmic radiation (SCR), at low altitude, mainly composed of muons and neutrons, with a small contribution of electrons and photons and a very small contribution of pions. Muons, electrons and charged pions form the charged particle fraction of SCR, whereas the uncharged fraction is composed of neutrons, photons, neutrinos and neutral pions. In aircraft cruising altitude, the composition is different, with a higher contribution of neutrons, protons, electrons and pions. For more details on the SCR cascades and references, see, e.g., [10] (p. 160). Here, we are especially interested in the muon component, called the “hard” component because of the high energy of muons (between 1 and 20 GeV; UNSCEAR 2000 [11], annex B, p. 86). The composition of SCR is strongly variable with altitude, see, e.g., Figure 30 of [12], which has consequences for the response characteristic of radiation monitors (see below). The intensity of SCR depends on several factors:

- Mass of air above the measurement point, related to air pressure, is in turn mainly controlled by altitude above sea level (a.s.l.) plus variations caused by meteorological variability. For estimating local SCR dose rate, usually altitude a.s.l. is taken as an approximate predictor. Ref. [13] gives the figure ΔADR/ADR ≈ −0.01564 Δp [kPa] for muons. For variations of 10 kPa (100 hPa; standard air pressure at sea level is 1013 hPa) due to weather variation, one thus finds about 15.6% ADR variation.
- Geomagnetic latitude: There is a latitude dependence of the SCR intensity caused by the geomagnetic shielding of primary cosmic rays before they enter the atmosphere. Therefore, the intensity of SCR at certain altitudes also depends on geomagnetic latitude. The effect origins from the fact, that to the geomagnetic field measured by the geographically variable, the so-called cut-off rigidity; e.g., Figure 2 in [14]. Geomagnetic and geographical latitudes coincide only very approximately; the lowest SCR intensity is found in the equatorial region, and the highest at high latitudes. The geomagnetic axis is the theoretical line between the geomagnetic poles, not to be confused with the magnetic poles. Both axes do not coincide with the geographical axis and moreover, the magnetic axis does not pass through the centre of the Earth. Geomagnetic coordinates are defined relative to the geomagnetic poles. The geomagnetic field appears distorted compared to the geometry of the globe (e.g., https://geomag.bgs.ac.uk/education/earthmag.html accessed on 4 November 2024). The SCR intensity difference between the equator and 60° N is about 10% at ground level [15], higher for higher altitudes. From figures given in (Table 2) in [16] one finds that the intensity in terms of dose equivalent rate is 6% and 10% higher at 55° N than 43° N at sea level and at 3 km a.s.l., respectively, during solar minimum. During solar maximum, the figures are 2% and 6%, respectively.
- Solar activity: Higher solar activity and resulting solar wind leads to repulsion of galactic cosmic rays. SCR intensity therefore follows the about 11-year solar activity cycle (which is itself modulated by longer-term cycles and overlaid by irregular variability components). During solar minima, SCR intensity at ground altitude can be up to 10% higher than during solar maxima. An irregular component is added by so-called Forbush events, which are sudden and short-term (lasting about a week) decreases in SCR due to solar coronal mass ejection. The last solar activity minimum occurred about 2019/2020 [17], and the last activity maximum in 2024, which is expected to extend to 2025. This type of solar event can produce protons with energy high enough that they generate particle cascades which can be observed at the ground level, called Ground Level Events or GLEs. These are short pulses which last about an hour, followed by a decay tail. GLEs are rare, and so far, we have made no attempt to detect them with our means.
- Seasonal effect: According to [13,18], “owing to temperature changes in the upper layer of the atmosphere, the muon production rises in summer and, thus, the mean path [length] to ground level increases”. Due to the short lifetime of muons, a longer journey to the ground in summer results in fewer muons reaching the ground. The variation amounts to about 3%. (The result is valid for northern temperate latitudes).

Wissmann [13] concludes that the contributions of the latter two effects on the uncertainty of ADR measurement is 6.9 nSv/h at ground level, unless SCR is actually known through particular measurement. To make the matter even more complicated, Ref. [19] noted that G-M probes as used in the German ADR network “seem to change their response to SCR with increasing altitude” (because of the changing composition of the SCR, the authors presume).

A European map of dose due to SCR is shown in the European Atlas of Natural Radiation [10], together with physical background information. Ref. [20] calculated doses due to cosmic radiation for the world population. Useful information is also in ICRU (2010) [21].

### 2.2. Response of G-M Detectors to Cosmic Radiation

#### 2.2.1. Response to Muons

ADR detectors respond to SCR characteristically according the type of given device. Usually, this is assessed by measuring during airplane or balloon ascents above sea or on sufficiently large lakes (see Section 3.2) in different altitudes. For some discussion of cosmic response, see Section 2.2.3 in [22] and references there.

Anisotropy in response to secondary cosmic radiation has been discussed by [23] (pp. 20–22). For the muonic component of SCR, which is the only one relevant in the context of G-M counters, it follows from the mentioned report (rearranging from its Equations (3.1-3) and (3.1-4)) that for cylindrical detectors with effective radius and length R and L, respectively, the ratio of response RResp (axis horizontal : axis vertical) is approximately

RResp ≈ (1.583 + Q)/(1 + 2Q),

with Q = R/L (shape factor). Note that “axis horizontal” and “axis vertical” are equal to the “vertical position” and the “horizontal position” in the terminology of this paper. We do not know whether the thin window of the pancake detector used in the bGeigie Nano and the CzechRad affects this reasoning.

The bGeigie Nano and CzechRad have Q = 1.756 and consequently, RResp ≈ 0.740; this means that in the vertical position (axis horizontal), the detector is 0.740 as sensitive to cosmic muons as in the horizontal position (axis vertical). The same considerations seem to apply for other charged particles, i.e., electrons and protons. In the horizontal position, there is very little difference, if at all, between the positions of the thin window pointing upwards or downwards. Therefore, the vertical position is recommended as standard, because it reduces the influence of the SCR on the total reading. Field measurements above a lake performed in autumn 2024 gave a preliminary empirical value RResp = 0.708 ± 0.010. Further experiments are planned; details of the experiments and results will be published elsewhere. We do not know how precisely the Spiers model [23] is applicable to the particular geometry and architecture (different materials, window) of the detector used in the bGeigie Nano and the CzechRad.

#### 2.2.2. Response of G-M Detectors to Neutrons

Neutrons, also part of cosmic radiation, contribute very little to G-M signals, although they are an important part of the total cosmic ray dose. The sensitivity to neutrons relative to photons is quantified by the so-called k_u_ value, which is in the order of 1% for the typical energies of cosmic neutrons in low altitudes, whose spectral peaks are at 1 and 100 MeV. References include [24,25,26,27,28].

### 2.3. Dependence of Secondary Radiation on External Factors

#### 2.3.1. Altitude Dependence

Although from a rigid physical point of view, SCR intensity depends on the air mass above, and hence on air pressure, approximate formulae of altitude dependence which make calculation simpler have been proposed. In particular, if survey data are evaluated retrospectively, air pressure is not available. Usual pressure variation due to meteorological difference induces a variability in SCR of several percent; see Section 2.1, first bullet.

The most quoted formula for the charged particle component Z (H*(10), nSv/h), mainly muons, which is of interest here, comes from Bouville and Lowder (1988) [15] after [16], and is also quoted in UNSCEAR 2008 [11], annex B, p. 233ff and [10], p. 161 (for the annual effective dose; here, recalculated nSv/h ADER, assuming that the effective dose is about equal to H*(10)):

Z = 6.542 exp(−1.649 h) + 25.349 exp(0.4528 h),(1)

h in km a.s.l., Z in H*(10) nSv/h. It has been derived from measurements in 1965 (near solar maximum, i.e., SCR minimum) at 50° N geomagnetic latitude. Apparently, an ionization chamber, [29] which responds differently from a G-M counter, has been used.

For the German GS-05 monitor, used in the Early Warning Network IMIS (www.bfs.de/EN/topics/ion/accident-management/bfs/environment/imis.html, accessed on 4 November 2024), the following mean altitude dependence has been estimated (G-M tube vertical, geometry very different from bGeigie Nano and CzechRad):

Z = 41.71 + 1.131 × 10^−2^ h − 2.08 × 10^−6^ h^2^ + 1.9 × 10^−9^ h^3^, h in meters. (2)

**Figure 1:**
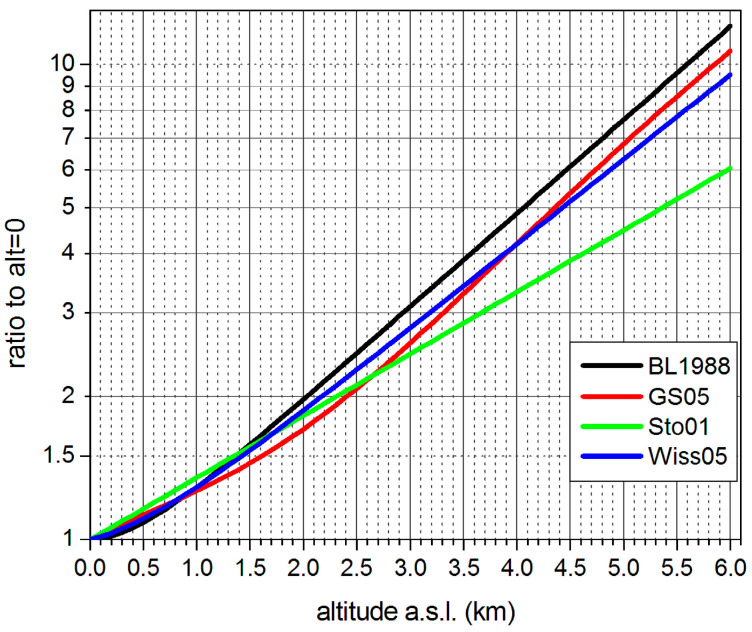
Approximate altitude variation in SCR intensity, charged component. For legend codes, see text.

**Figure 2:**
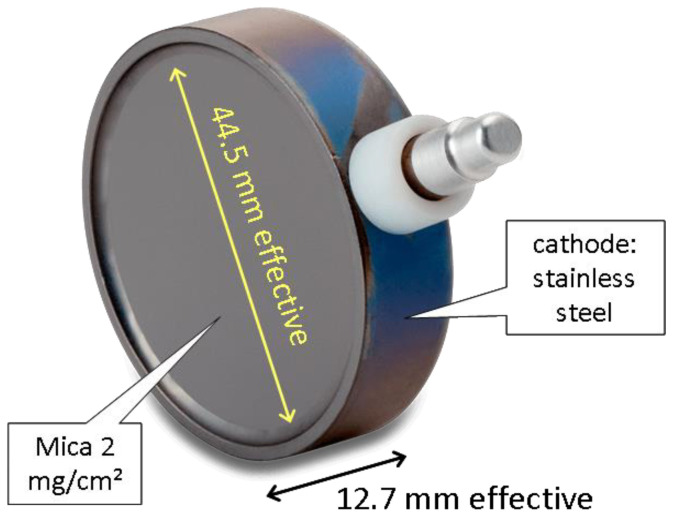
LND 7317 Pancake G-M detector. Picture from the manufacturer’s web page.

The approximate altitude effect for the charged component is shown in Figure 1. It is calculated from data which underlay Figures 2 and 3 in [22]. BL1988 refers to [15], Wiss05 to measurements in [18] using the GS05 monitor of the German ADR network, GS05 refers to the above formula derived from data of this monitor and Sto01 refers to a simplified formula for the same purpose (both only internally published; internal background, Section 2.5.1, subtracted). For low altitudes below about 2.5 km a.s.l., the values coincide rather well.

#### 2.3.2. Dependence on Solar Activity

Since solar activity modulates cosmic particle flux, one can expect SCR intensity to follow the same pattern. However, according to UNSCEAR 1982 [11] (graph in annex B, p. 85), the effect is very small at the ground level. On the other hand, it is notable in aircraft cruise altitude according to UNSCEAR 2000 [11] (graph in annex B, p. 88). At sea level, variation in dose rate has been estimated to be higher, namely about 10% over the 11-year solar cycle [13,15].

Cinelli et al. [30] investigated the variation in SCR at ground level between 2002 and 2015. The database includes selected stations of the EURDEP system in the far North of Europe or high in the mountains. For each year, the minimum was chosen which coincides with the thickest snow cover (strongest shielding of terrestrial radiation). The ensemble mean Y, standardized to the 2012 value, was fitted with a sinusoid,

y = A sin[(2π/11)(t − φ)],(3)

t in decimal years, A = 0.037 ± 0.016 (i.e., an amplitude 3.7%), phase φ = 2004.96. This empirical model is significant, *p* < 0.01, but possibly not optimal. y is positively correlated with neutron flux (Oulo neutron monitor data, https://cosmicrays.oulu.fi/ accessed on 4 November 2024; r^2^ = 0.52) and ^7^Be concentration in air (REM-db sparse network, [31], r^2^ = 0.25) is negatively correlated with sunspot number (https://www.sidc.be/SILSO/home accessed on 4 November 2024; r^2^ = 0.44).

#### 2.3.3. Dependence on Geographical Latitude

As explained above, the relevant latitude is the geomagnetic, but not the geographical one. However, the geomagnetic latitude is rarely known in practice; therefore, the geographical one is used as an approximation. The geomagnetic latitude of a location can be calculated from observation data. But geomagnetic coordinates move considerably fast against the geographical grid, so that using geomagnetic coordinates is impractical. On the internet, several facilities for calculating geomagnetic from geographical coordinates for a given time are available, such as [32] and [33]. The dose rate is about 10% lower at the geomagnetic equator than at high latitudes (UNSCEAR 2008, [34] Annex B; [15]).

### 2.4. The bGeigie Nano and CzechRad Monitors

The bGeigie Nano monitor has been developed by the Safecast team since 2011 in several stages. The current version has been the standard instrument for about 7 years. In the following years, it has also been adopted as the standard device in the Czech Republic in project “RAMESIS” of the national radiation protection authority [35,36,37,38], which aims to equip citizens, schools and the like with radiation monitors and relatively easy to use free QGIS (version 3.16 and higher with current version of Radiation Toolbox plugin) [39] as part of environmental education. Similar attempts have been on the way in Slovakia [40].

In the Czech Republic, the device was replaced by an upgraded version called CzechRad in 2023, but the same detector was used, following the same philosophy [41]. A better GPS module is used and other minor technical modifications have been applied.

The bGeigie Nano has been subjected to QA in dosimetric laboratories, and its behaviour in a well-controlled metrological environment is moderately well documented [42,43].

According to https://github.com/Safecast/bGeigieNanoKit/wiki/Specifications (accessed on 4 November 2024), the G-M detector is an “LND Inc. Type 7317 Pancake Halogen-Quenched Geiger-Mueller (G-M) tube, 2”; Effective diameter 1.75” (45 mm); Mica window density 1.5–2.0 mg/cm^2^. It is capable of detecting Alpha, Beta and Gamma radiation. It comes with a Medcom iRover controller and HV (high voltage) supply. Its accuracy is ±10% typical, ±15% maximum, and its allowable temperature range is −20° to +50 °C.” More information is given by the manufacturer, www.lndinc.com/products/geiger-mueller-tubes/7317 (accessed on 4 November 2024), from where the picture is taken (Figure 2).

Unfortunately, calibration of the bGeigie Nano is not very well documented. The only document available to us is [42]. In an experiment performed at Jülich Research Center (Germany), a bGeigie Nano (complete in its case) was positioned in a ^137^Cs gamma beam, once facing the beam (“horizontal position”), then perpendicular to it (“vertical”). Different true dose rates were applied. For the lowest (with 10 μSv/h still high), the calibration factors were 361 and 238 cpm/(μSv/h) for horizontal and vertical position, respectively (calculated from the figures given there). It is not clear whether the µSv/h denotes the ambient dose equivalent rate (ADER), H*(10), as it should be in Europe. But since the described experiment has been performed in Jülich (Germany), it can be plausibly assumed. (The generic quantity is denoted ADR, while the values denote ADER because of the calibration to it; similar to the quantity “length”, measured in “meters”.)

The ratio of sensitivities between horizontal and vertical positions equals 1.60 (for low doses), i.e., the detector is 60% more sensitive in the position facing the beam, than perpendicular to it. However, it must be emphasized that this is true only for the experimental setup of the laboratory using a narrow beam, but not for field conditions. In these, the detector is exposed to an area source, namely the ground containing gamma radiating radionuclides, above which it is positioned (plus cosmic rays and Rn progeny, to be discussed separately).

In https://github.com/Safecast/bGeigieNanoKit/wiki/Specifications (accessed on 4 November 2024), a calibration factor 334 cpm/(μSv/h), corresponding 35.9 (nSv/h)/(counts per 5 s), is quoted for ambient radiation, i.e., from area sources in contrast to point sources. This is the factor which is implemented in the bGeigie Nano firmware and which is used in the Safecast map. We do not know how this factor has been determined, to which detector orientation it refers and whether it denotes ADER. According to our own investigations, variability (1 SD) between exemplars of the device is about 4%. Calibration by the Safecast team was carried out with a ^137^Cs source, and thus refers to 662 keV. This made sense because the bGeigie Nano was initially conceived for surveying contamination in the region affected by the Fukushima fallout. Today, detectors for environmental surveys are preferably calibrated with ^226^Ra, whose spectrum better resembles ambient gamma ray spectra. Also, the CzechRad was calibrated with ^137^Cs. However, since the weighted mean energy of typical ambient spectra is close to that of ^137^Cs, these differences are not very important.

Regarding gamma rays, impulses recorded by a G-M counter result mainly from the interaction of photons with the material of detector case. Muons also interact directly with the gas filling the detector. Electrons are largely absorbed by the detector case. The exact physical processes which lead to a certain response of the detector to incident radiation are quite complicated.

Counting cycles are 5 s, which for typical mean ambient radiation levels (1–4 counts/5 s) leads to rather high uncertainty. Therefore, sliding means over 12 cycles, i.e., 1 min is used in the map but the counts/5 s are also stored on the SD card. These were used in our evaluations. Again, according to our investigations, the internal clocks of the bGeige Nanos are a bit inaccurate and the counting cycle deviates slightly from 5 s, but differently for each exemplar so far investigated. The mean cycle length of 14 analyzed exemplars of bGeigie Nano is 5.015 ± 0.009 s (1 SD), or a deviation of 0.28% from the nominal 5 s. This effect, although very small compared to other uncertainty components and irrelevant in practical usage, adds to sample deviation in the measured sensitivity between bGeigie Nano exemplars. It also makes comparison of parallel operating monitors difficult because after some time they are out of pace. The CzechRad monitors seem to have a more accurate timer. Further, the detectors tend to produce spurious count numbers occasionally, that is, counts within one separated cycle which cannot be explained by Poisson statistics or very short external pulses. The occurrence frequency is different between detectors [5].

### 2.5. Components of Ambient Dose Rate Readings

#### 2.5.1. Internal Background

Even without external radiation hitting the detector, it will record pulses. Their sources are traces of radionuclides within the detector material and electronic noise. The most common way to determine this internal background (also called intrinsic BG or zero effect) is measurement within lead shields (usually coated with thin layers of Cd and Cu to shield K_x_ fluorescence rays from Pb and Fe) located in deep underground salt mines. Salt contains practically no natural radionuclides; remaining gamma radiation is almost completely shielded by the lead. The depth below the surface shields from most cosmic muons. World-wide, several underground laboratories of the kind exist. The method can be applied for the bGeigie Nano only by turning it on while a GPS signal is available; it will continue recording if the signal disappears. If it is turned on in absence of a GPS signal, it will measure but not record. The feature complicates its use underground to some extent. This issue has been solved better for the CzechRad, which creates an extra file if no GPS is available.

#### 2.5.2. Measurement in Absence of the Terrestrial Component

An alternative is measurement in situations without terrestrial radiation, when only BG and cosmic components that contribute to the count rate are present. Experiments have to be performed to separate these components. Some experiments are described in this section. For a more thorough discussion of ADR components, see, e.g., [22] and references there.

Several sets of bGeigie Nano and CzechRad data measured above water were analyzed; see Section 2.6.2. If the water body, such as a lake, sea or large river, is deep enough, practically at least 3 m, and the measurement is performed far away from the shore, practically at least 100 m, the ADR measured is composed of contributions of

+ internal background of the device (BG)

+ cosmic dose rate (DR)

+ dose rate produced by radionuclides contained in the boat plus (in some cases) persons, possibly attenuated by shielding

+ dose rate from radionuclides in water

+ dose rate from radionuclides in the air.

The water component can be assumed to be negligible. The contribution of cosmogenic radionuclides suspended in air deposited on the ground (mainly ^7^Be and ^22^Na) is below 1 nSv/h; see [22] and references there.

#### 2.5.3. Radon Progeny

The air component, due to radon progeny (mainly ^214^Pb and ^214^Bi), depends on the proximity of solid ground that exhales Rn. Over sea, the air component is practically zero (e.g., [44] report ~10 μBq/m^3^ over the equatorial Pacific Ocean), while on lakes, it can amount to a few up to a few 10 nSv/h, depending on the geological nature of the surrounding land (hence, Rn exhalation from the ground) and meteorological conditions. Factors of the latter are height of the atmospheric mixing layer, wind speed and soil humidity, which affects Rn transport in the ground and thus the exhalation rate. Typical in-land ^222^Rn concentration is 10 Bq/m^3^ (ADER about 3 nSv/h), while over granite and ground with high gas permeability and thus high Rn exhalation, 20 Bq/m^3^ (ADER ≈ 6 nSv/h) and higher are possible. Locally, 100 Bq/m^3^ (ADER ≈ 30 nSv/h) have been reported. The values can be assumed representative for geographically similar areas. For a literature review on outdoor Rn surveys, see [45].

Dose conversion factors (DCFs) of ^214^Pb and ^214^Bi for submersion in a semi-infinite air volume are, according to ICRP 144 [46], (p. 105; H*(10)): 0.416 and 0.076 (nSv/h)/(Bq/m^3^), respectively, or 0.492 in sum. EPA (2019), [47], p. 211, for adults: 0.26 and 0.04, respectively, or sum 0.30; Previous sources [48]: 0.5 for EEC; DOE (1988) [49]: effective dose (recalculated from obsolete units): 0.25 and 0.029, respectively, or sum 0.28; [50], p. 196: 0.236 and 0.043, respectively, or sum = 0.33. Here, we propose using the value of 0.5 with outdoor equilibrium factor F = 0.6 (UNSCEAR 2000, [34] annex B, §213, p. 203), 10 Bq/m^3^ of ^222^Rn yield 10 × 0.6 × 0.5 = 3 (nSv/h)/(10 Bq/m^3^). Ref. [51] found F = 0.34 to 0.62, mean 0.48 in a German survey; an overview of equilibrium factors: [52]. However, we think that uncertainties of DCF and F are of minor importance compared to the estimation of local Rn concentration.

### 2.6. Determination of the Internal Background and Cosmic Response

#### 2.6.1. Method I: Aircraft Ascent and Descent

bGeigie Nano monitors were carried on several airplane journeys within Europe. The routes are shown in Table 1. In all cases, the monitors were in vertical orientation (axis of the detector horizontal) and positioned close to a window. The journeys took place between autumn 2022 and summer 2024, i.e., close to the solar activity maximum (late 2024–early 2025) [17], which means a low intensity of SCR. Radiation by aircraft components, passengers and cargo, as well as attenuation by aircraft components, is unknown and has not been considered. The same applies to gamma radiation from radon progeny in the outside atmosphere, but this component is probably very low, below 1 nSv/h we assume. Above 0.5 km above ground, the terrestrial gamma component can be neglected.

It should be emphasized that measured dose rates do not represent the doses received by persons in the airplane, because the monitors are not calibrated for cosmic radiation. This applies in particular to higher altitude, since the composition changes with altitude.

In addition, to investigate the latitude effect, we evaluated the measured dose rate by GPS latitude. This was done separately in ascent/descent and in cruising phases of flights. Cruising altitude is typically 10–13 km. To compare the values between flights, they were normalized as

Y_norm_ := Y/Y_0_, Y_0_ (4)

Y_0_ according to the third-order polynomial of Figure 4.

The normalized response refers to the vertical position of the monitor. All data are for high solar activity.

#### 2.6.2. Method II: Measurement Above Water Bodies in Different Altitudes

Let Y(raw) be the measured ADR values (ADER in nSv/h). ‘BG’ denotes the internal background (which shall be estimated), Rn denotes the ADR component caused by gamma rays from Rn progeny, ‘cosm × f’ denotes the adjusted cosmic component (see below) and ‘other’ denotes any other possibly contributing components, such as persons present during measurement (a very small effect, if at all), remaining terrestrial gamma radiation and gamma radiation of the platform on which the experiment takes places.

Then, the model reads

Y(raw) = BG + Rn + cosm × f + other.(5a)

If sufficiently many measurements Y(raw) are available and ‘Rn’ and ‘other’ can be approximately guessed, ‘BG’ and the cosmic response can be estimated by regression.

Assumptions:

- All measurements were performed with detectors which have the same BG. This is not exactly true in reality, but one has to live with this uncertainty, about 10%.
- Outdoor radon concentration can be guessed approximately from experiences about mean Rn concentration in different geographical regions. The value is subject to meteorological variability (specifically, height of the atmospheric mixing layer), of which we have no control, but which we can guess to vary by factors (0.1, 5) and more. Many studies have been conducted about temporal variability in outdoor Rn concentration. References include UNSCEAR (1988) [11] (Annex A, §85ff), [53,54,55,56,57,58,59,60,61,62,63,64,65,66,67,68]. It would be worthwhile to further evaluate the literature to refine estimation of the ADR component due to Rn’s levels dependence on measurement season and time.
- For the cosmic component, we use the estimated value of the GS-05 G-M counter in the vertical position (used in the German Early Warning Network) in the northern temperate latitude according to the formula given in [3], Equation (2) and the Bouville–Lowder formula (Equation (1)). Factors f are applied: for low latitudes, cosmic dose rate is assumed 10% lower; if the value Y(raw) by bGeigie has been measured with the detector horizontal (axis vertical), f = 1.412 (see Section 2.3). No distinction between window facing up or down is made at this stage. The uncertainty of cosm × f is probably 10% at most.

The regression model can then be rewritten:

Y(raw) − Rn − other =: Y = BG + b × (cosm × f).(5b)

b denotes the response of the bGeigie relative to GS-05. “other” denotes the dose rate component from nearby objects, such as a boat or a bridge, on which the experiment is performed or by the presence of persons who contain gamma radiating ^40^K.

The quantities to be estimated are hence BG and b. Y is the dependent and (cosm × f) the independent variable.

## 3. Results and Discussion

We report two types of experiments: (1) Evaluation of the readings of bGeigie Nano monitors during flights in commercial aircraft; (2) Evaluation of measurements above water bodies. Although most experiments were performed with bGeigie Nano monitors, the results also apply to the CzechRad due to its very similar architecture, although it uses slightly different electronic hardware parts.

### 3.1. Measurements in Aircraft

#### 3.1.1. Dose Rate by Altitude

**Figure 3:**
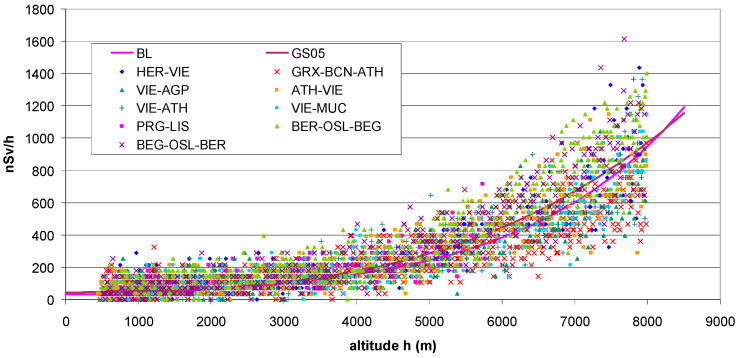
Dependence of dose rate recorded with bGeigie Nano detectors on flight altitude above see level (GPS data). Flights according to Table 1. Theoretical values from the Bouville–Lowder formula (BL; Equation (1)) and the GS05 are added (Section 2.3.1; Equation (2)).

**Figure 4:**
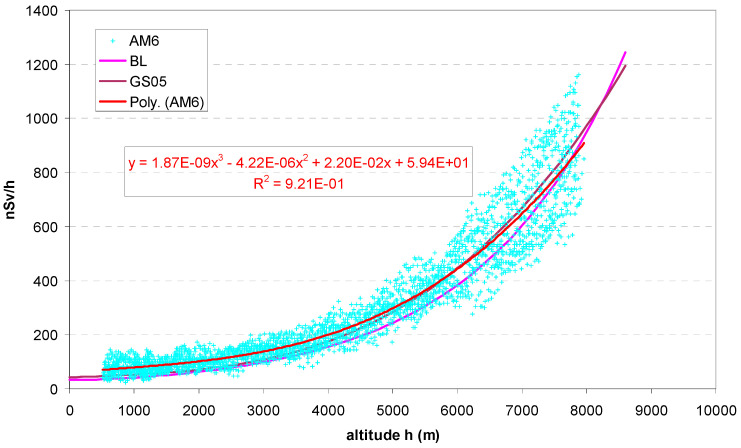
All values (30 s running averages) plotted in one graph, and regression curve (polynomial of order 3). Only values measured at least 400 m above ground and below 8000 m used.

The values measured during the evaluated flights (Table 1) are shown in Figure 3. The results appear essentially consistent between the flights. Dispersion and data noise are still high. Apart from statistical fluctuations, systematic reasons may be as follows: different attenuation by the aircraft shell; radiation of the aircraft, the passengers and other objects; variations in outside air pressure which distort the relationship with altitude; the latitude effect (Section 2.3.3), since the journeys took place between about 35° N (Heraklion) and 60° N (Bergen). The variation due to the solar cycle is probably low since all flights took place near maximum solar activity. To reduce noise, a 6-point running average was applied, corresponding to 6 × 5 s = 30 s average values labelled AM6 is shown in Figure 4 together with a fitted polynomial of order 3. (The fit to the original, non-averaged data is very similar).

**Figure 5:**
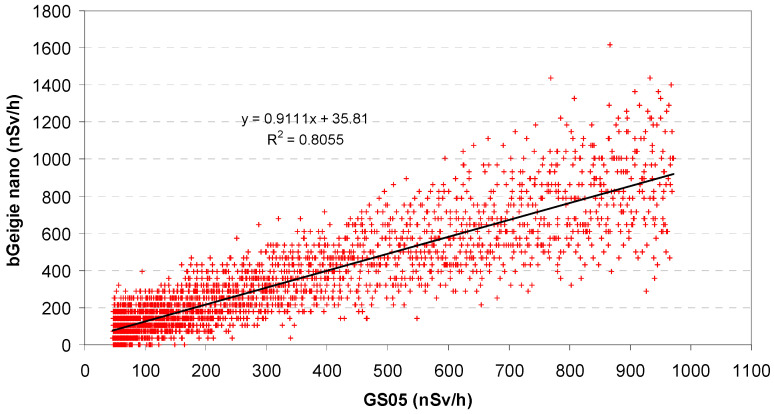
Correlation between recorded bGeigie Nano values and theoretical values for a GS05 monitor.

The values measured with the bGeigie Nano can be compared with the theoretical values from the Bouville–Lowder formula and the GS05 (Equations (1) and (2), see Section 2.3.1) at the same altitude. Figure 5 shows a quite good linear correlation with the GS05 monitor. The correlation with the values according to BL is worse and visibly non-linear (not shown here; it can well be approximated with a quadratic function). Evidently, G-M detectors react to cosmic radiation differently from ionization chambers, although the curves look very similar in the previous figures.

#### 3.1.2. Latitude Effect

**Figure 6:**
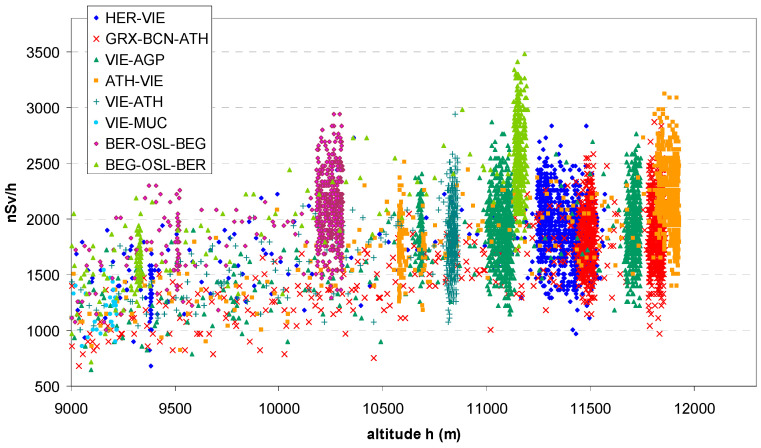
Identification of cruising altitude from clusters formed by dose rate data vs. GPS altitude.

**Figure 7:**
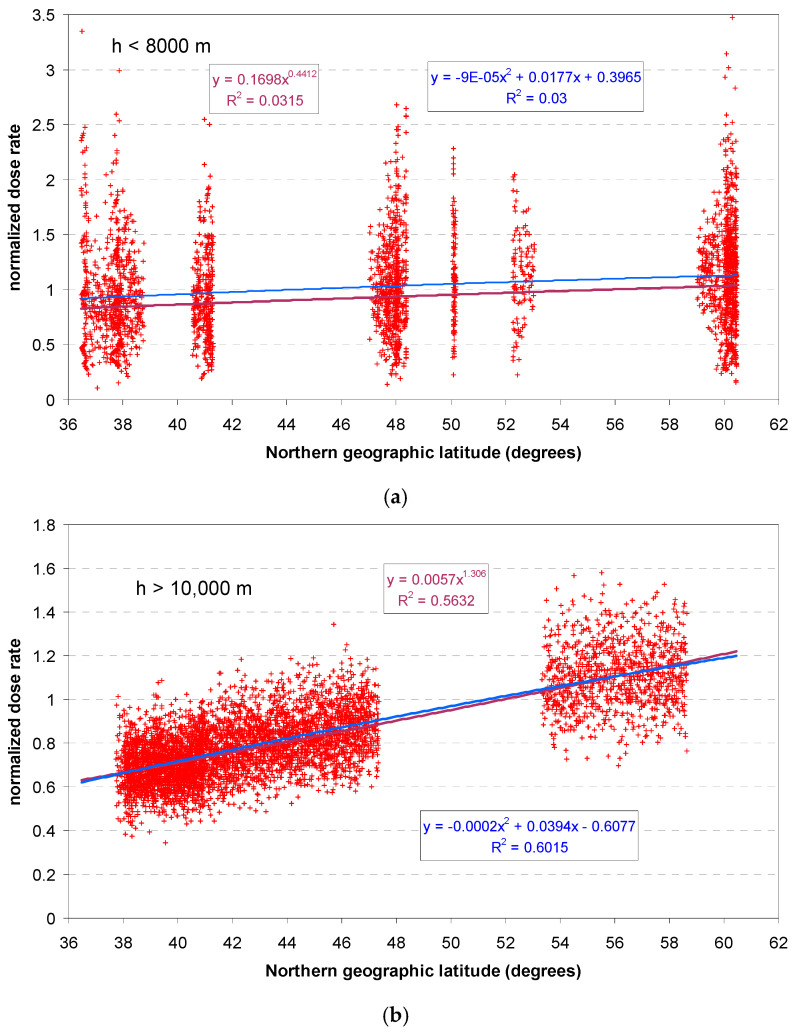
Scatter plots of normalized dose rate vs. geographic latitude. (**a**) Flight altitude < 8000 m, (**b**) >10,000 m.

From the individual dose rate vs. altitude scatter plots and GPS altitudes, approximate cruising altitudes were identified according to the clusters, Figure 6, summarized in Table 1. Plots of the normalized response Y_norm_ are shown in Figure 7a, for flight altitudes below 8000 m, corresponding to the ascent and descent phases, and above 10,000 m, corresponding to cruise altitude, in Figure 7b.

The latitude effect is visible in both cases, but more distinct in high altitude > 10 km. The effect is not linear with altitude. Therefore, a power model and a second-degree polynomial have been fitted. These fits are purely empirical and have no physical rationale. The stronger effect in high altitude can be seen from the higher exponent (power model) and the higher first-order term (polynomial model). It would be worthwhile to investigate the effect of altitude in more detail, but the database is not sufficient for this.

### 3.2. Measurements Above Water Bodies

Sources of the data used for the analysis are shown in Table 2 and in more detail in Appendix A. Rn contributions are very rough estimates in the absence of measured values. An uncertainty of 50% is assumed; it may be even higher. For the equilibrium factor F, we set 0.6 and DCF = 0.3 (Section 2.5.3). Other contributions (Section 2.6.2): very rough guesses, based on the visual assessment of the exposure situation, uncertainty 50% assumed.

**Figure 8:**
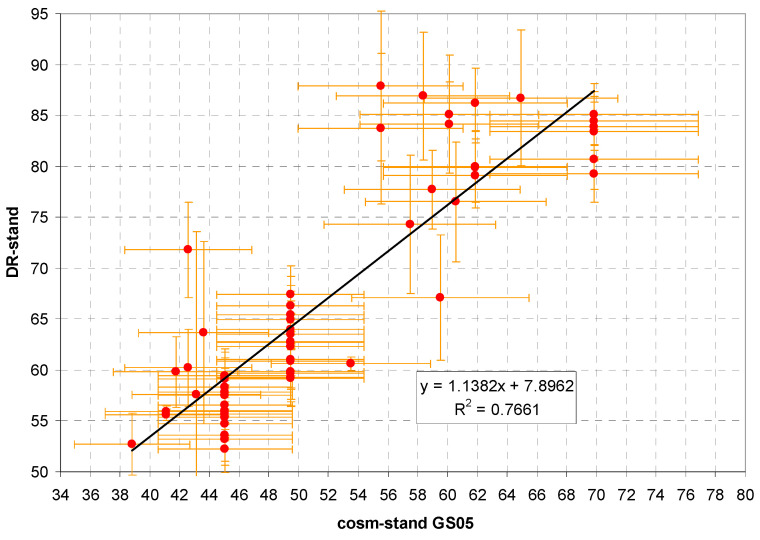
Linear regression of the standardized measured dose rate, DR-stand (nSv/h) against standardized theoretical GS05 dose rate (nSv/h).

The resulting regression plot of standardized measured dose rate against the theoretical dose rate according to the GS05 monitor following the model given in Section 2.6.2 is shown in Figure 8. x-axis uncertainty is chosen as 10%, lacking better knowledge. Detailed results of different regression models performed with Past 4.17 software [69] are given in Table 3.

While correlation is clearly visible, the data points are strongly scattered. Reasons for this may include incorrect estimates of certain input parameters such as Rn concentrations, variation in the latitude factor or variation in parameters between individual devices, for example, RResp or the internal BG.

**Figure 9:**
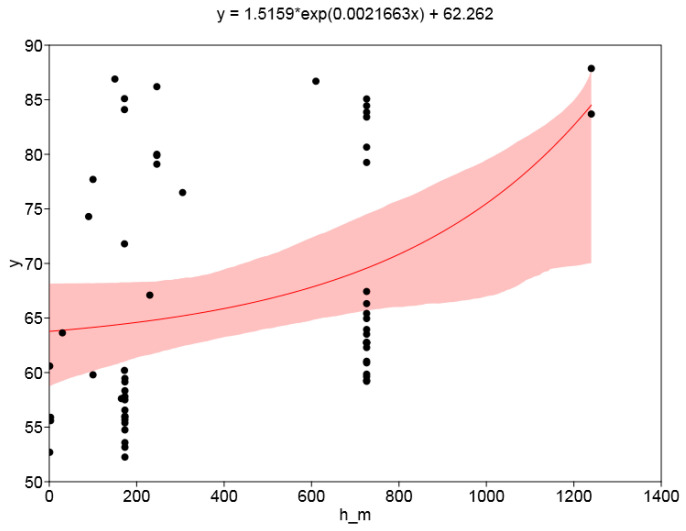
Fitted exponential model. x-axis: altitude (m a.s.l.), y-axis: standardized measured dose rate (nSv/h). Shaded area: 95% confidence interval.

The scatter plot of measured dose rate vs. altitude appears very dispersed, Figure 9. Fitting exponential models with Past 4.17 software results in the following parameters:

model y = a exp(bx): a = 62.14 nSv/h, b = 0.000181 m^−1^, r^2^ = 0.12 (6a)

model y = a exp(bx) + c (shown in Figure 9): a = 1.516 nSv/h, b = 0.002166 m^−1^; c = 62.26 nSv/h; r^2^ = 0.13; a + c = 63.78 nSv/h. (6b)

## 4. Conclusions

**Figure 10:**
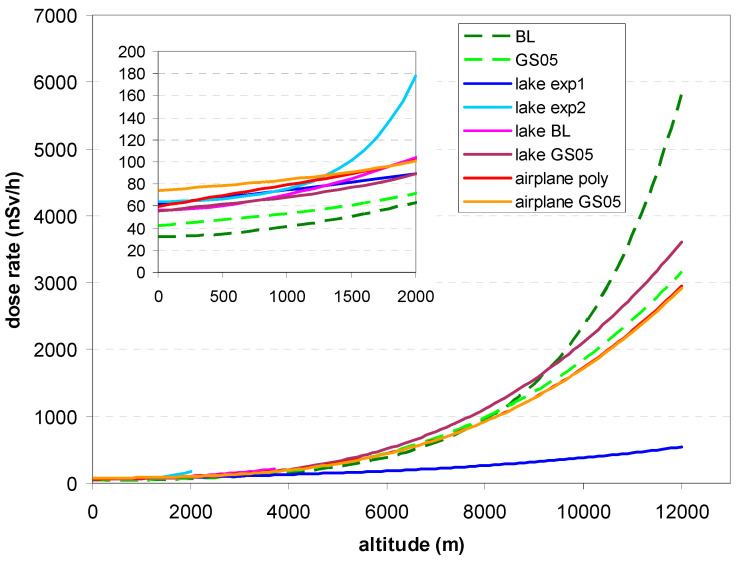
Comparison of different models of response of the bGeigie Nano to cosmic radiation. Dashed curves: Bouville–Lowder (BL) formula and GS05 model. BL: Equation (1); GS05: Equation (2); Lake BL, GS05: from Table 3; Lake exp1 and lake exp2: models Equation (6a,b); Airplane poly, GS05: Figure 4 and Figure 5.

(1) Essentially, the response of the bGeigie Nano and CzechRad monitors to altitude is as could be expected for G-M detectors. Two types of experiments, which, however, both rely on measuring in different altitude above sea level, lead to qualitatively similar results.

(2) Different functions of the bGeigie Nano’s response to cosmic radiation, estimated from data, are plotted in Figure 10. One can see that for low altitude, models derived from the lake experiments “lake exp 1” (Equation (6a)), “lake BL” and “lake GS05” (Table 3) perform similarly, as does the model “airplane poly” (Figure 5). For higher altitude, both models derived from airplane experiments (Figure 4 and Figure 5) perform about equally.

The functions represent means over northern latitudes typical for Europe. For an approximate latitude correction, one can use the results of Figure 7. But apparently the latitude effect increases with altitude: our data are not sufficient to evaluate this additional effect.

All results apply to high solar activity. Approximate adjusting could be made using Equation (3). However, it has been derived for low altitude measurements and also, in this case, we do not know its altitude dependence. (Equation (3) has been used in the evaluation of the lake experiments, Section 2.6.2 and Section 3.2).

(3) A reasonable estimate of the internal background is 10 nSv/h, although variation between individual devices must be expected. Estimation of individual BGs was not possible with the experiments reported here.

We plan to continue these experiments, including more exemplars of bGeigie Nano and CzechRad. In particular, the anisotropic response of pancake detectors to cosmic radiation (Section 2.2.1) shall be further investigated, as well as the influence of Rn progeny. Also, a detailed characterization of the CzechRad including a calibration report is planned to be published later. Regarding the context of Citizen Science and practical usage, we are thinking of a guide about measurement QA, including measurement protocols and addressing the errors which result from deviation from protocols. Also, formulae for accounting for the effects discussed in this paper and data interpretation would be part of such a guide. We may also extend the investigation to other types of ADR monitors, such the Radiacode, which is increasingly popular in the Citizen Science context and which is based on a scintillation detector.

## Figures and Tables

**Table 1:** Dose rate measurements during flights.

Flights	Dates	Monitor #	Approximate Cruising Altitudes (km)
Berlin (BER) ↔ Oslo (OSL) ↔ Bergen (BEG)	September 2022	3273	9.32; 9.51; 10.18–10.30; 11.14–12
Prague (PRG)–Lisbon (LIS)	October 2022	3281	GPS failed at cruising alt.
Athens (ATH) ↔ Vienna (VIE)	May–June 2023	3273	10.80–10.87; 10.58; 11.83–11.93
VIE–Munich (MUC)	November 2023	3273	9.1–9.2
VIE–Malaga (AGP)	June 2024	3273	10.68–10.7; 11–11.3; 11.68–11.74
Granada (GRX)–Barcelona (BCN)–ATH	June 2024	3273	11.4–11.5; 11.78–11.85
Heraklion (HER)–VIE	July 2024	3273	9.38; 11.24–11.53

# indicates Identification number of the devices, both for bGeigie Nano and CzechRad.

**Table 2:** Data used for evaluation of lake experiments. Altitude: meters a.s.l; hor/ver: horizontal or vertical orientation of the monitors; Rn contribution: Bq/m^3^. Other contributions: 1 nSv/h, if not stated otherwise.

Location	Altitude (m)	hor/ver	Remarks; Assumed Rn Concentration
Pacific Ocean, 2019	1	ver	Rn: 0
Helicopter ascent above Mediterranean, Southern France, 2019	90–610	hor	Rn: 0; other: 2; 5 altitude steps, 1 measurement each
Sea off Costa Rica, 2020	1	hor	Rn: 0
Lake Balaton, Hungary, 2020	100	both	Rn: 7; 2 measurements
North Sea off coast, Germany, 2020	3	ver	Rn: 1; 2 measurements
Danube bridge, Vienna, Austria, 2020	172	both	Rn: 7; other: 3; 4 measurements
Danube ferry crossing, near Vienna, 2020	165	ver	Rn: 7
Frozen lake near Prague, Czech Republic, 2021	246	hor	Rn: 10; 4 measurements
Lipno lake, Southern Czech Republic, 2021	726	both	Rn: 15; 22 measurements
Bridge above Hardanger Fjord, Norway, 2022	30	ver	Rn: 5; other: 3; possible interference by adjacent rocks
Bortolan lake, Poços de Caldas, Brazil, 2023	1240	both	Rn: 10; other: 2; 2 measurements
Lhota lake near Prague, Czech Republic, 2024	173	both	Rn: 10; 14 measurements

**Table 3:** Regression results for the linear model standardized bGeigie Nano measurements vs. theoretical results for GS05, Equation (2) and Bouville–Lowder (B-L) formula, Equation (1).

Statistic	x = stand. GS05	x = stand. B-L
Ordinary least square:		
intercept	7.90 ± 4.40	7.22 ± 4.16
slope	1.138 ± 0.083	1.538 ± 0.105
Orthogonal (RMA):		
intercept	−0.5 ± 4.4	−0.3 ± 4.2
slope	1.300 ± 0.083	1.731 ± 0.105
r^2^; p (both):	0.77; 1.2 × 10^−19^	0.79; 6.8 × 10^−21^

**Figure A1:**
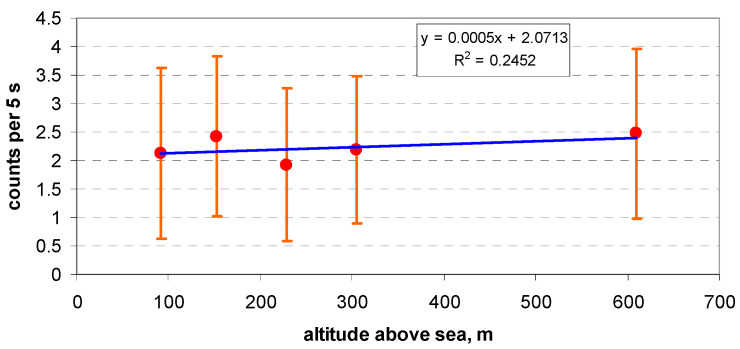
bGeigie readings in a helicopter at different altitudes. Arithmetical mean and standard deviations.

## Data Availability

Some data are available on justified request.

## Abbreviations

ADR	Ambient dose rate
ADER	Ambient dose equivalent rate
AM, SD, SE	arithmetical mean, standard deviation, standard error
a.s.l.	above sea level
BG	background
CS, CM	Citizen Science, Monitoring
cpm	counts per minute
DCF	dose conversion factor
G-M counter	Geiger Müller counter
QA	Quality Assurance
SCR	Secondary cosmic radiation

## Appendix A

Experiments for determining internal background and cosmic response: some additional information about the experiments mentioned in Table 2, Section 3.2.

*Pacific Ocean crossing by ship, 2019*

In a previous paper, [3], the following estimate was performed (text taken from the paper):

A very approximate preliminary estimate of the internal BG (also called self effect or intrinsic BG) can be gained the following way: A bGeigie Nano detector was used during a crossing of the Pacific Ocean by sail ship (https://safecast.org/lessons-from-a-record-setting-pacific-crossing-with-safecast-on-board/; accessed on 4 November 2024). The observed ADER consists of BG + cosmic radiation + some contribution of the structural material of the ship and radiation emitted by people (^40^K mainly). Assuming 41.7 nSv/h as ADER on sea level and 53.7 ± 12.7 (1 SD) nSv/h as observed during the ocean crossing, we find a difference of 12 nSv/h. Accounting for a possible contribution of ship and crew, about 1 nSv/h, we assume an internal BG corresponding to approximately 11 nSv/h.

*Off the coast of Costa Rica, 2020*

Measurements on a glass fiber boat off the Pacific coast were performed with a bGeigie Nano in horizontal position, with the window facing downwards. Again, for ADER at sea level + internal BG + possible contribution of boat and persons, 61.1 ± 14.5 nSv/h was found. Location: 8.7° northern latitude, 83.3° western longitude.

*Tidal area of the North Sea, 2020*

A bGeigie Nano was fixed on a buoy not far from the North Sea shore and the dose rate was measured during high tide. We assume an Rn concentration of 1 Bq/m^3^ close to the shore. The remaining terrestrial radiation and the metal structure on which the monitor was suspended may add 1 nSv/h.

The experiment was performed by Dr Christoph Ilgner, Ministry for Environment, Agriculture and Energy of Saxony-Anhalt State in Magdeburg, Germany. The location is 53.91° northern latitude, 8.67 eastern longitude, near Cuxhaven, Germany.

*Lake Balaton, Hungary, 2020*

Measurements were performed on a pedal boat made of plastic, rented at the public beach of Balatonalmádi, summer 2020. The measurement location was about 500 m off the shore. However, the lake is quite shallow, probably less than 3 m, so that some residual terrestrial radiation must be expected, for which together with the boat plus passengers we guess 1 nSv/h. The location is 47.03° northern latitude, 18.02° eastern longitude.

*Measurements from bridges, 2020 and 2022*

In summer 2020, measurements were performed while crossing two bridges over the Danube and the New Danube in Vienna, Nordsteg and Jedleseer Brücke, respectively. The main Danube river is at least 300 m wide, which guarantees sufficient distance to the terrestrial environment. The New Danube is a bit less than 200 m wide, so that some terrestrial radiation influence can be expected. The Nordsteg bridge (48.25° northern latitude, 16.38° eastern longitude) is made of concrete, which yields a radiation background due to building material, for which we guess 3 nSv/h. Jedleseer Brücke (48.27° northern latitude, 16.37° eastern longitude) is essentially a steel construction with some asphalt pavement. Its radiation background can be expected to be low; as a guess, we set it to 3 nSv/h. Both rivers are >3 m deep, and the bridges are about 10 m above water surface. Altitude a.s.l. is 172 m. For Rn concentration, we guess 7 Bq/m^3^.

Two passages in a bus over the Hardangerbrua, a large suspension bridge over the Hardanger Fjord, Norway, in September 2022, were also evaluated. The bridge is over 50 m above the water level, and the fjord is several 100 m deep. Distance to the shores is sufficient. Radiation by bridge and bus may contribute 3 nSv/h. For Rn, we guess 5 Bq/m^3^. The location is 60.40° northern latitude, 5.32° eastern longitude.

*Danube ferry, 2020*

Dose rate was measured during the crossing of the Danube near Vienna on a small wooden passenger/bicycle ferry. The Danube is wide enough and some meters deep. The crossing takes about 15 min, but only data from a small section in the middle of the river were used for evaluation. The location is 48.33° northern latitude, 16.33° eastern longitude.

*Frozen lake near Prague, 2021*

Dose rate was measured at a small lake in a park at the outskirts of Prague, location 50.04° northern latitude, 14.54° eastern longitude. Altitude a.s.l. is 246 m. For Rn concentration, we guess 10 Bq/m^3^ [70]. For possibly remaining terrestrial and other components, we set it to 1 nSv/h.

*Helicopter ascent, 2019*

A bGeigie Nano was operated during a helicopter flight above the Mediterranean Sea whose purpose was QA of professional instruments. The experiment was performed at about 43.2° northern latitude, 4.9° eastern longitude. Measured ADR is composed of secondary cosmic radiation, internal background, gamma radiation of the helicopter and personnel and gamma radiation emitted by Rn progeny in the air.

Measurements were performed 300, 500, 750, 1000 and 2000 feet above sea level, or about 90, 150, 230, 305 and 610 m. The detector was positioned horizontal (axis vertical) with the window facing downwards. The results shown here are based on 64 to 69 measurement cycles of 5 s, in each altitude. As Figure A1 shows, the differences between altitudes are very small, which is no surprise since altitude variation is also small. To estimate response at sea level, regression was performed for a linear and exponential model; the approximation is fair for small altitude differences. Both models yield an intercept 2.07, with SE 0.18 for the linear model. The slope (5.5 × 10^−4^ (counts/5s per m) for the linear model) is not significantly greater than zero. With calibration 334 cpm/(μSv/h), one finds 74.4 nSv/h for sea level. However, feeding the data into the more comprehensive model shown in Section 2.2 leads to more reliable results. No relevant dose rate contribution due to Rn is expected in this case. For the remaining component (helicopter body, persons), we set it to 2 nSv/h.

*Lake Lipno in S Bohemia, summer 2021*

Two sets of bGeigie Nano monitors (two boxes containing 8 and 4 units) were used for measurements in a site with about 8 m water depth and >500 m distance from the shore. Measurements with vertical and horizontal orientations of the detectors were performed. One monitor failed, so that there were (8 + 4 − 1) × 2 = 22 measurements. The location is 48.7° northern latitude, 17.1° eastern longitude, 726 m a.s.l.

*Lake Lhota, near Prague, Czech Republic, autumn 2024*

On lake Lhota near Prague, several bGeigie Nano and Czechrad monitors were exposed on a raft away from the shore in vertical and horizontal position. The main purpose was examination of the difference between vertical and horizontal orientations of the monitors. Details of the experiments will be published elsewhere. Counting times about 30 min. The location is 50.27° northern latitude, 14.67° eastern longitude, altitude = 173 m a.s.l.

*Lake Bortolan, Poços de Caldas, Brazil, November 2023*

A similar experiment has been performed using two bGeigie Nano devices on the lake Represa Bortolan as part of a radiometric exercise, November 2023 [71]. The location is 21.79° southern latitude, 46.64° western longitude, 1240 m a.s.l. In this case, the carrier was a rented pedal boat. Counting times were between 6 and 12 min only, which resulted in higher uncertainty than for the Lhota lake experiment, although on the other hand, the higher altitude meant a higher count rate.
